# Moderating effects of depressive symptoms on the relationship between problematic use of the Internet and sleep problems in Korean adolescents

**DOI:** 10.1186/s12888-018-1865-x

**Published:** 2018-09-04

**Authors:** Min-Hyeon Park, Subin Park, Kyu-In Jung, Johanna Inhyang Kim, Soo Churl Cho, Bung-Nyun Kim

**Affiliations:** 1 Department of Psychiatry, St. Paul’s Hospital, College of Medicine, The Catholic University of Korea, Seoul, Republic of Korea; 2 National Center for Mental Health, Seoul, Republic of Korea; 3 Department of Public Medical Services, Seoul National University Bundang Hospital, Bundang, Sungnam-Si Republic of Korea; 4 Department of Psychiatry, Korea Armed Forces Capital Hospital, Bundang, Republic of Korea; 5 Division of Child and Adolescent Psychiatry, Department of Psychiatry and Behavioral Science, Seoul National University College of Medicine, 101 Daehak-No, Chongno-Gu, Seoul, Republic of Korea

**Keywords:** Insomnia, Excessive daytime sleepiness, Depression, Sleep-wake rhythm

## Abstract

**Background:**

Adolescence is a period of marked sleep pattern changes and sleep problems, which may result from both endogenous and exogenous factors. Among the various factors affecting adolescent sleep, depression and problematic Internet use (PIU) have received considerable attention. We examined if there is a different PIU effect on sleep between depressed group and non-depressed groups.

**Methods:**

Data for a total of 766 students’ between 7th and 11th grades were analyzed. We assessed various variables related sleep to problems and depression and compared those variables between an adolescent group with problematic Internet use (PIUG) and an adolescent group with normal Internet use (NIUG).

**Results:**

One hundred fifty two participants were classified as PIUG, and 614 were classified as NIUG. Compared with the NIUG, the members of the PIUG were more prone to insomnia, excessive daytime sleepiness and sleep-wake behavior problems. The PIUG also tended to include more evening types than the NIUG. Interestingly, the effect of Internet use problems on sleep problems appeared to be different according to the presence or absence of the moderating effect of depression. When we considered the moderating effect of depression, the effect of Internet use problems on sleep-wake behavior problems, insomnia and excessive daytime sleepiness increased with increasing Young’s Internet Addiction Scale (IAS) scores in the non-depressed group. However, in the depressed group, the effects of Internet use problems on sleep-wake behavior problems and insomnia did not change with increasing Internet use problems, and the effect of Internet use problems on excessive daytime sleepiness was relatively decreased with increasing Internet use problems in the depressed group.

**Conclusions:**

This study demonstrated that the effect of PIU on sleep presented differently between the depressed and non-depressed groups. PIU is associated with poorer sleep in non-depressed adolescents but not in depressed adolescents. This finding might be observed because PIU may be the biggest contributor to sleep problems in the problematic Internet user without depression, but in the problematic Internet user with depression, depression might be a more important contributor to sleep problems; thus, the influence of PIU on sleep effect might be diluted.

## Background

The prevalence of sleep problems in adolescents is very substantial; the prevalence of insomnia in adolescents is as high as 20~ 30% [1, 2], and approximately 16% of high school students suffer from excessive daytime sleepiness [3]. Sleep problems during adolescence may result from both endogenous and exogenous factors and some endogenous reasons for sleep problems are very common in adolescents, such as primary insomnia, insomnia related to depression and delayed-phase sleep patterns, which occur according to changes in the biological clock [4, 5]. Some sleep problems in adolescents, especially short sleep duration, are commonly induced by external factors, such as both increased academic pressure and electronic device use time including the Internet [6]. Among the various factors known for their relationship with adolescent sleep problems, depression and problematic Internet use (PIU) have received considerable attention.

It has been reported that the incidence of depression is increased in adolescents who sleep less than 6 h per day [7], and regardless of sleeping hours, impairment of sleep quality itself is related to depression, excessive daytime sleepiness and decline in academic performance [8]. Furthermore, it is known that inadequate sleep and impairment of sleep quality can affect not only the severity of depressive symptoms but also suicide ideation [9]. Not only can sleep problems cause depression, but it also true that depression can cause sleep problems [10, 11]. Approximately, 75% of individuals with depression suffer from a lack of sleep and poor sleep quality [12], and the typical sleep pattern of individuals with depression is characterized by decreased slow -wave sleep, short rapid eye movement (REM) sleep latency, long duration of first REM sleep, and increased density of REM sleep [13]. A total of 72% of children with major depressive disorder have sleep disturbances, 53.5% have insomnia alone, and 10.1% have both disturbances [14].

It is known that PIU is related to sleep problems such as reduce sleeping time [15, 16], the tendency to sleep later [17], sleep disturbance [18], insomnia [19], an increased level of tiredness [20], and excessive daytime sleepiness and poor school performance [21–25]. Furthermore, exposure to the bright light of a screen until late at night may suppress melatonin secretion and sleep-wake phase-delay, and may increase alertness [26, 27], which can cause sleep induction problems [28, 29]. Many terms have been used to describe the phenomenon characterized by an individual’s inability to control their Internet use leading to marked distress and/or functional impairment [21] such as PIU, compulsive Internet use, Internet addiction, Internet dependence, pathological Internet use and so on. Among these concepts, we decided to use “PIU” in this paper, because we wanted to explore more general and common Internet- use problem rather than the minimum numbers of extreme pathologic Internet usage. It is also well known that compared with non-depressed adolescents, depressive adolescents show a high prevalence of PIU and vice versa [30] .

Interestingly, around the world South Korean adolescents are notorious for a prevalence of depression and PIU and the shortest sleep duration on weekdays [31]. Therefore, we aimed to examine whether depressive symptoms moderate the effect of PIU on sleep in South Korean adolescents, a group that is very characteristic and famous for a high prevalence of these three problems. Thus, we hypothesized that PIU acts differently on sleep between adolescents in the depressed group and non-depressed groups.

## Methods

### Participants

Participants were recruited from one middle school and one senior high school located in Seoul, South Korea. After the school principals approved the research, the investigators visited the schools, explained the purpose of the study to students and teachers, obtained consent. We also mailed letters to parents to outline the study’s objectives, guarantee confidentiality, provide a contact telephone number for the principal investigator, and to indicate that parents would be informed of the results after the analyses were completed. The letter also included a statement that parents were free to refuse to respond if they did not agree with the study’s objective. The Institutional Review Board (IRB) for Human Subjects at Seoul National University Hospital approved the study protocol. Detailed information about the study was given to parents and children, and written informed consent was obtained before study entry.

Among the students of these schools, a total of 802 students between 7th and 11th grade (age range: 12–17 years old) volunteered to participate in this study. Parents were asked to complete questionnaires about socio-demographic characteristics (e.g., family income and, parental educational level) and parenting attitudes, which were returned after 3 days. For each student, all of the study scales were administered in 1 day in December. Therefore, we were able to successfully match the season among participants. Of the 802 students who participated in the study, 36 were excluded due to incomplete responses, which resulted in a total of 766 subjects (483 boys, 283 girls). The mean age ± standard deviation (SD) of the 766 subjects was 15.05 ± 1.35 years.

### Measures

#### Young’s Internet addiction Scale (IAS)

The IAS is a self-reported 20-item scale that ranges from 1 to 5 points for each item. An IAS score of 40–69 indicates that problems with the use of the Internet are occasional or frequent. A score of 70 or higher is considered a serious problem with Internet use. In a previous study of the IAS, the instrument’s split-half reliability was 0.729 and its Cronbach’s alpha was 0.713 [32]. We set the cut–off value for PIU at 40 and dichotomized the respondents as either problematic Internet use group (PIUG) or normal Internet use group (NIUG).

#### Insomnia Severity Index (ISI)

The ISI was developed by Morin and colleagues [33] and has been used for the last 20 years. It is a 7-item scale assessing the perceived severity of insomnia symptoms (initial, middle, and terminal), degree of satisfaction with sleep, interference with daytime functioning, awareness of impairment, and concern caused by sleep problems [34]. Respondents use a 5-point Likert-type scale ranging from 0 to 4 to describe their insomnia over the past 2 weeks. In a previous study of the ISI, there was a high degree of internal consistency, with Cronbach’s alpha of 0.92 [35].

#### Epworth Sleepiness Scale (ESS)

Daytime sleepiness was measured using the Epworth Sleepiness Scale (ESS), a frequently used subjective 3-point sleepiness scale that rates the likelihood of dozing in 8 daily life situations [36]. Higher scores in the ESS represent a greater propensity for sleepiness. In the present study, excessive daytime sleepiness was defined as an ESS score of ≥8. The Korean version of the ESS has been verified as a reliable and valid measure of daytime sleepiness [37] and is commonly used for adolescents [3]. The internal consistency of the questionnaire in healthy controls was fair with a Cronbach’s α value of 0.73. For patients, the Cronbach’s α was 0.90, higher than the previously reported value of 0.88, indicating a high level of internal consistency [37].

#### The Children’s Depression Inventory (CDI)

The Children’s Depression Inventory (CDI) is a 27-item, self-rated, symptom-oriented scale that is suitable for youth aged 7 to 17. Each CDI item consists of three statements that are rated on a scale that ranges from 0 to 2. The total score ranges from 0 to 54 [38, 39]. Based on previous study results, we set the cut-off value for depression at greater than or equal to 16. Therefore, we classified the participants who score equal to higher 16 in the CDI as depressed group and the participants who scored lower than 16 as non-depressed group. In a previous study of CDI, there was a high degree of internal consistency, with the Cronbach’s alpha of 0.88 [40].

#### The School Sleep Habits Survey (SSS)

The School Sleep Habits Survey asks about usual sleep/wake patterns over the previous 2 weeks and includes a Sleepiness Scale, a Sleep-Wake Behavior Problems Scale, a Depressed Mood Scale, and a Morningness-Eveningness scale [41].

In this study, we used Sleep-Wake Behavior Problems Scale, and Morningness-Eveningness scale among the four subscales because the Sleepiness Scale measures same construct as the ESS and the Depressed Mood Scale measures the same construct as the CDI.

The Sleep-Wake Behavior Problems Scale contains 15 items that reflect a combination of difficulties with sleep initiation and maintenance, as well as other sleep-related problems (e.g., nightmares); a higher score indicates more sleep problems. Scores range from 10 to 75, and the alpha coefficient for the sleep/wake behaviors scale was 0.75 [41].

The Morningness-Eveningness scale assesses sleep preference according to the adolescent’s sleep–wake proclivity. This scale consists of 10 questions about the preferred timing of activities, such as waking time, taking tests, and bedtime. Scores range from 10 to 42, with higher scores indicating a morning chronotype [41–43]. The morning and evening groups were operationally defined as the participants who scored in the top and bottom 25% of this scale, respectively. This operational definition method was used in a previous study [44].

### Statistical analysis

The chi-square test and Student’s t-test were conducted to compare categorical and continuous variables, respectively, between the groups (PIUG vs NIUG, depressed group vs non-depressed group). Hierarchical regression analyses were conducted to determine the associations between the independent variables (if an individual is categorized as PIUG or NIUG, or the IAS scale scores) and dependent variables (the scores of sleep-related scales). We used the Baron and Kenny method to examine the moderating effects of depressive symptoms between the independent and dependent variables [45]. Significance for all tests was set at *p* < 0.05. All statistical analyses were performed using SPSS 20.0 for Windows (SPSS Inc., Chicago, IL, USA).

## Results

Among the total 766 participants who completed the questionnaires, 152 were categorized as PIUG and 614 as NIUG. Table 1 shows the differences in demographic and clinical characteristics between the NIUG and PIUG. Compared with the NIUG, the PIUG had significantly higher ISI, ESS, and SSS_ Sleep-Wake Behavior Problems Scale scores (*p* < 0.0001, *p* < 0.0001, and *p* = 0.001, respectively). Compared with the NIUG, the PIUG had a significantly lower Morningness-Eveningness scale total score (*p* = 0.021), and the ratio of morning types, as classified by their total Morningness-Eveningness scale scores, was significantly lower in the PIUG than in the NIUG (25.57% VS 15.13%, *p* = 0.007). The mean bedtime of the PIUG was significantly later than that of the NIUG, both on weekdays (*p* = 0.006) (Table 1). The reason for the later bedtime in the PIUG is that 3.3% of the NIUG and 11.3% of the PIUG went to bed late due to computer or smartphone use (*p* = 0.005) on weekdays and 39.1% of the NIUG and 45.3% of the PIUG went to bed late due to computer or smartphone use (*p* = 0.189) on weekends. Overall, 62.6% of the NIUG and 82.0% of the PIUG answered that they use smartphones or computers immediately before falling asleep (*p* < 0.0001). The mean weekend wake up time was significantly later in the PIUG than in the NIUG (*p* = 0.001). However, although the mean weekday wake up time tended to be later in the PIUG, no significant difference was detected between the two groups (*p* = 0.757).

**Table 1:** Demographic and clinical characteristics of adolescents with and without problematic Internet use

	Adolescents without PIU (*N* = 614)	Adolescents with PIU (*N* = 152)	*p*
Age	14.93 ± 1.28	15.60 ± 1.51	< 0.0001
Gender (female)	260 (42.34%)	23 (15.13%)	< 0.0001
Mean computer use time on weekdays (S.D.)	1.42 ± 1.56	2.56 ± 2.14	< 0.0001
Mean computer use time on weekends (S.D.)	2.59 ± 2.72	4.97 ± 3.79	< 0.0001
Mean smartphone using time during weekdays (hours per day) (S.D.)	3.51 ± 3.43	4.27 ± 3.42	0.015
Mean smartphone use time on weekends (S.D.)	4.58 ± 4.13	5.53 ± 4.47	0.014
Total sleep duration on weekdays	7:10 ± 1:39	6:48 ± 1:32	0.006
Total sleep duration on weekends	9:17 ± 2:08	9:11 ± 2:10	0.572
Mean bedtime on weekdays	23:49 ± 1:26	24:08 ± 1:05	0.004
Mean bedtime on weekends	24:16 ± 1:31	24:52 ± 1:35	< 0.0001
Mean wake up time on weekdays	6:59 ± 1:02	6:58 ± 0:56	0.757
Mean wake up time on weekends	9:42 ± 1:44	10:12 ± 1:58	0.001
Insomnia severity index total	8.96 ± 4.14	10.43 ± 4.31	< 0.0001
Epworth sleepiness scale total	6.30 ± 3.96	7.69 ± 4.52	< 0.0001
School sleep habit survey: Sleep-wake behavior scale total	11.92 ± 6.67	14.00 ± 8.26	.001
School sleep habit survey: morningness-eveningness (M-E scale) scale total	25.89 ± 3.68	25.11 ± 3.58	0.021
Ratio of evening type by M-E scale	152 (24.76%)	46 (32.26%)	0.179
Ratio of morning type by M-E scale	157 (25.57%)	23 (15.13%)	0.007
Children’s depression inventory total	14.70 ± 7.60	18.70 ± 8.64	0.001

*PIU* problematic Internet use, *M-E* morningness-eveningness

Additionally, compared with the NIUG, the PIUG showed significantly higher CDI score (*p* = 0.001). The depressed participants (CDI score equal to greater than 16) had higher ISI scores (9.90 ± 4.44 vs 8.69 ± 3.92, *p* = 0.003) and lower Morningness-Eveningness scale scores (24.12 ± 4.25 vs 26.28 ± 3.36, *p* < 0.001) than the non-depressed participants. The ratio of evening to morning types was significantly higher in the depressed group (73.19%) than in the non-depressed group (38.32%, *p* < 0.0001).

Among the 152 individuals in the PIUG, 109 (71.74%) were classified as the depressed group in the PIUG. No differences were found in sleep-related scores (ISI, ESS, SSS_ Sleep-Wake Behavior Problems Scale, and Morningness-Eveningness scale scores) and sleep-related habits (mean wakeup time, mean bedtime and total sleep duration on both weekdays and weekends) between the depressed and non-depressed groups in the PIUG.

**Fig. 1:**
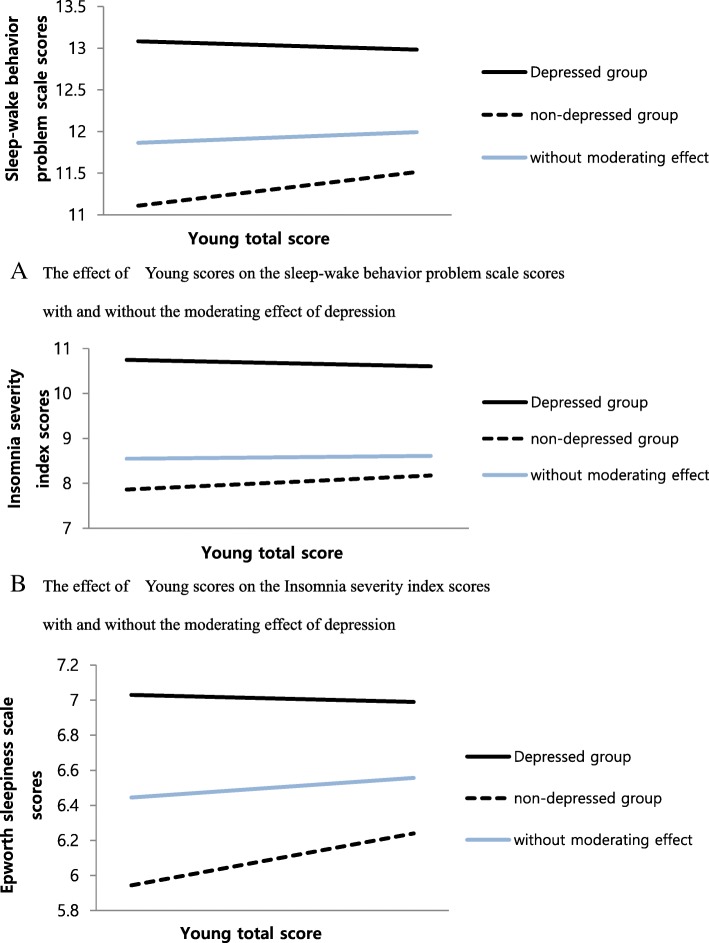
The moderating effect of depression on the relationship between IAS scores and sleep-problem scale scores. **a** The effect of Young scores on the sleep-wake behavior problem scale scores with and without the moderating effect of depression. **b** The effect of Young scores on the Insomnia severity index scores with and without the moderating effect of depression. **c** The effect of Young scores on the Epworth sleepiness scale scores with and without the moderating effect of depression

Additionally, we examined the moderating effect of depressive symptoms on sleep–related problems (insomnia, excessive daytime sleepiness, and sleep-wake behavior problems) with PIU using the Baron and Kenny method. In step 1, we conducted a regression analysis in which we analyzed the effects of the IAS scores and whether the individuals were included in the depressed group. Then, in step 2, we input the interaction variable between the IAS scores and whether the individuals were included in the depressed group back into the step 1 analysis. The step 1 analysis revealed that PIU did not have an effect on the SSS_Sleep-Wake Behavior Problems Scale (*p* > 0.05) or ISI (*p* > 0.05). However, in step 2, we found a moderating effect of depressive symptoms on the SSS_ Sleep-Wake Behavior Problem Scale and the ISI scores (Fig. 1). In the depressed group, the SSS_ Sleep-Wake Behavior Problems Scale and ISI scores were not increased even with increasing IAS scores, but in the non-depressed group, the SSS_Sleep-Wake Behavior Problem Scale and ISI scores increased with increasing IAS scores. The effect of IAS scores on ESS scores was different, depending on whether the individuals were depressed. In the depressed group, the ESS score was decreased with increasing IAS scores, but in the non-depressed group, the ESS score was increased with increasing IAS scores.

## Discussion

The results of this study show that compared with the NIUG, PIUG members were more prone to insomnia, excessive daytime sleepiness and sleep-wake behavior problems. The PIUG also tended to include more evening types than the NIUG. Interestingly, the effect of Internet use problems on sleep problems appeared to be different according to the presence or absence of depressive symptoms.

PIU is associated with poorer sleep in nondepressed adolescents but not in depressed adolescents. In detail, the effect of PIU on sleep-wake behavior problems, insomnia and excessive daytime sleepiness increased with increasing IAS scores in the non-depressed group. However, in the depressed group, the effects of PIU on sleep-wake behavior problems and insomnia did not change with increasing Internet use problems, and the effect of Internet use problems on excessive daytime sleepiness was relatively decreased with increasing Internet use problems in the depressed group. This result suggests that when a person has depressive symptoms and PIU at the same time, the two factors might act differently rather than exhibit additive, mutually reinforcing effects. However, we failed to identify the underlying causes of these patterns due to the cross-sectional nature of this study and the lack of observable differences in sleep problems between the depressed and non-depressed groups in the PIUG. A previous study that examined the effect of insomnia and Internet addiction on depression showed that both Internet addiction and insomnia affect depression, but exert distinct effects [19]; when insomnia is an explanatory factor, insomnia strengthens the negative effects of Internet addiction on depression, and when Internet addiction is an explanatory factor, Internet addiction strengthens the negative effects of insomnia on depression.

The Morningness-Eveningness type is well-recognized as a reflection of the endogenous biological clock. Morning types prefer performing intellectual and physical activities during the morning, and evening types prefer performing activities during the late afternoon or evening [43]. Compared to morning types, evening types tend to subjectively feel that their sleep time is not enough during weekdays and have increased sleep time during the weekend due to sleep debt accumulated through the weekdays [43]. Compared to morning types, evening types tend to report subjectively poor sleep quality and excessive daytime sleepiness [44]. Morning types tend to have healthier life-styles than evening types [46], and evening types tend to have higher depression rates than morning types [47]. In this study, the PIUG tended to include more evening types than the NIUG, and the depressed group also tended to include more evening types than the non-depressed group. However, interestingly, within the PIUG group, there was no difference in the Morningness-Eveningness type between the depressed and the non-depressed groups. Generally, it is expected that self-induced sleep restriction in adolescents would aggravate the changes in the sleep-wake cycle and therefore cause delayed sleep phases and lack of enough sleep [48]. However, this study result suggests that if an individual adolescent has clinical-range PIU and depressive symptoms, depressive symptoms and PIU act independently of sleep problems rather than mutually initiating a vicious cycle.

During adolescence, there is a dramatic change in sleep as a normal developmental process. As adolescents get older, they tend to have delayed sleep-wake phase, less sleep time and sleep architectural changes, such as declines in slow-wave sleep and slow-wave activity [48]. While the large part of these sleep changes across adolescence are shown to be a consequence of normal brain development in adolescence, more prevalent causes of sleep problems, especially short sleep duration, resulted from exogenous reasons in South Korean adolescents [49, 50]. South Korean adolescents are famous for their short sleep duration around the world [51, 52]. National statistics show that the average sleep duration of high school students is as short as 5 h 27 min in Korea [53]. Strong cultural emphasis on academic performance in South Korea and the high rate of PIU among Korean adolescents (12.5%) [54] are two strong exogenous reasons for this short sleep duration [6]. According to an international survey, Korean adolescents recognize themselves as the most unhappy adolescents in the world and also have a high prevalence of depression [55].

Sleep is essential for mental and physical development and daily function in adolescents [7, 56–58]. Therefore, it is very important for adolescents to secure adequate sleep time and quality. Although understanding the underlying causes of adolescent sleep problems is critical for solving the problems, it is not easy to identify the cause of individual adolescent sleep problems because adolescent sleep problems may arise from the interactions of various factors, such as PIU, depression and normal physiological and hormonal changes. Therefore, the collaboration of experts in related areas is critical for exploring the causes in a more multi-dimensional way.

The limitations of this study include the following. 1. Due to the cross-sectional study design, a causal relationship cannot be examined. Therefore, caution is needed when interpreting or generalizing the current findings. 2. PIU and depressive symptoms are unlikely to be the only two explanatory factors of sleep disturbance. Other covariates may include the sleep environment, anxiety, dietary ingestion, etc., and those factors were not evaluated.

## Conclusion

This study demonstrated that the effect of PIU on sleep presented differently between the depressed and non-depressed groups. PIU is associated with poorer sleep in non-depressed adolescents but not in depressed adolescents. This finding might be observed because PIU may be the biggest contributor to sleep problems in the problematic Internet user without depression, but in the problematic Internet user with depression, depression might be a more important contributor to sleep problems; thus, the influence of PIU on sleep effect might be diluted.
